# Early Vascular Damage in Young Women with DM-1 and Its Relation to Anti-Müllerian Hormone: A Cross-Sectional Study

**DOI:** 10.1155/2016/1487051

**Published:** 2016-08-29

**Authors:** Annelien C. de Kat, Hendrik Gremmels, Marianne C. Verhaar, Frank J. M. Broekmans, Felicia Yarde

**Affiliations:** ^1^Department of Reproductive Medicine, University Medical Center Utrecht, Heidelberglaan 100, 3508 GA Utrecht, Netherlands; ^2^Department of Nephrology and Hypertension, University Medical Center Utrecht, Heidelberglaan 100, 3508 GA Utrecht, Netherlands

## Abstract

Vascular function is suggested to be associated with ovarian reserve, but the relationship with microvascular function has never been studied. In this cross-sectional pilot study, the relationship of microvascular damage markers with AMH was studied in premenopausal women. Twenty-two regularly cycling women with type 1 diabetes (DM-1) and a reference group of 20 healthy regularly cycling women were included, from whom blood was drawn in the early follicular phase of the menstrual cycle. The main outcome was the correlation between circulating progenitor cells (CPCs), markers for early vascular damage, and AMH, a marker for ovarian reserve. Secondary endpoints for early vascular impairment were circulating angiogenic cells and additional biomarkers. Median AMH levels were 2.2 *µ*g/L [1.2–3.5] in the DM-1 group and 2.1 *µ*g/L [0.85–3.8] in the reference group. CPCs were significantly decreased in women with DM-1; 1204 ± 537 CD34+/CD45dim cells were counted in the DM-1 group, compared to 2264 ± 1124 in the reference group. CPCs and other markers of early vascular damage were not correlated with AMH levels in a multivariable analysis. These results underscore previous findings of early vascular damage in DM-1 and suggest that there may not be a relationship between vascular function and ovarian reserve.* Trial Registration*. This trial is registered with Clinicaltrials.gov NCT01665716.

## 1. Introduction

Menopause is the term used to describe the end of a woman's reproductive lifespan, following at least twelve consecutive months of amenorrhea [1]. The ovarian aging process that dictates the onset of menopause results from a gradual decrease in both quantity of oocytes and their quality, altogether referred to as ovarian reserve [2]. Besides chronological age, genetic, lifestyle, and environmental factors are thought to influence the pacing of ovarian aging [2, 3]. However, current knowledge of contributing factors to the decline of ovarian reserve remains scarce and determinants of the variation in rate of decline and age at menopause still remain to be elucidated.

In the postmenopausal state, there is an increased risk of cardiovascular disease [4]. This effect is associated with age at menopause [5] and is more profound in women with premature menopause (<40 years), suggesting a cardiovascular protective role of the premenopausal endocrine environment [6]. Levels of anti-Müllerian hormone (AMH), a marker that expresses ovarian reserve quantity [7, 8], were furthermore negatively correlated with atherosclerosis development and plaque size in a prospective primate study [9]. However, premenopausal cardiovascular risk was associated with age at menopause in a study with data from the Framingham Heart Study, leading to the hypothesis that the onset of menopause may be influenced by cardiovascular risk status, rather than the reverse [10]. This hypothesis is supported by a study in cynomolgus monkeys, where a diet-induced atherogenic lipid profile was associated with a decrease in follicle numbers [11].

Type 1 diabetes mellitus (DM-1) is associated with an increased risk of microvascular and macrovascular complications, which hold a strong association with endothelial dysfunction [12]. Endothelial function is of influence in several aspects of cardiovascular disease, including atherosclerosis progression [13]. Women with DM-1 have been shown to exhibit increased menstrual cycle disturbances and amenorrhea [14] and decreased AMH levels above the age of 33 compared to healthy controls, stratified for age and body mass index (BMI) [15]. They are furthermore reported to have fewer offspring compared to age-stratified controls [16, 17], and it is a matter of debate whether they enter menopause earlier [18, 19]. This, in turn, raises the question of whether an adverse vascular environment in diabetes could cause a reduction of ovarian reserve. Recently, the Ovarian Aging in Type 1 Diabetes Mellitus (OVADIA) study examined the relationship between macrovascular function tests and AMH levels in women with Type 1 Diabetes Mellitus (DM-1) [20]. While no association was found between macrovascular functions in DM-1 and AMH, the microvascular environment remained unexplored [20].

Microvascular function can be assessed by quantifying the number of circulating progenitor cells (CPCs) and circulating angiogenic cells (CACs). CPCs are circulating cells derived from the bone marrow and are important contributors of repair of vascular injury and neovascularization [21, 22], as well as predictors of future cardiovascular events [23]. CACs are cultured cells from the mononuclear cell fraction which fulfill an angiogenic purpose and were shown to be decreased in patients with cardiovascular disease, compared to healthy controls [24, 25]. CPCs and other markers of endothelial dysfunction were previously found to be lower in patients with DM-1 [26–28] but have never been studied in relation to markers of ovarian reserve.

In the current study, we aimed to examine whether microvascular, rather than macrovascular, function is related to ovarian reserve. To this end, we assessed microvascular function and ovarian reserve in a subset of both women with DM-1 of the OVADIA study and healthy controls.

## 2. Materials and Methods

### 2.1. Study Population

The current study was conducted as a pilot study within the OVADIA study (registered under trial number NCT01665716 at Clinicaltrials.gov). Women with DM-1 aged 18 to 45 years registered at the University Medical Centre Utrecht (UMCU) or other participating hospitals in the region were included in the OVADIA study. Each participant who was included for the OVADIA study between September 2012 and March 2013 and was available for further inclusion was additionally included in the current pilot study. After each inclusion of a participant, a female health care employee of the UMCU with the same age (difference of <12 months) and OC use status (current user; yes or no) was included, serving as a healthy reference. All participants were premenopausal and regularly cycling women. In the case of OC users, a reported regular cycle before the start of OC was considered sufficient for inclusion. Women with a history of surgery or radiotherapy to the pelvic organs, chemotherapy, menstrual cycle disturbances, or a suspicion of polycystic ovary syndrome (PCOS) were excluded. In addition, exclusion criteria for the reference group were chronic medication use or a diagnosis of any active disease at the time of inclusion.

### 2.2. Ethical Approval

Ethical approval was granted by the Institutional Review Board of the UMCU.

### 2.3. Study Design

Participants gave written informed consent. All participants received a questionnaire with questions regarding their general medical history, reproductive history, menstrual cycle, smoking status, medication use, and family history of age at menopause. 15 mL venous blood was drawn in the early follicular phase of the menstrual cycle, that is, within the first three days following menstruation. Women using OC were examined between day 5 and day 7 during their pill-free week. CPC and CAC numbers as well as AMH levels were determined from this blood sample and plasma was stored for determination of additional markers of vascular damage. In the DM-1 group, hemoglobin A1c (HbA1c) was additionally measured after an overnight fast.

### 2.4. Circulating Progenitor Cells

CPCs were identified by the presence or absence of cell surface antigens CD34, CD133, CD45, and KDR. CD34+/CD45dim cells were considered to be the primary CPC subset. The antibody panel consisted of the following antibodies: 2 *µ*L FITC mouse anti-human CD34 (BD Pharmingen; NJ, USA), 2 *µ*L phycoerythrin conjugated mouse IgG anti-hVEGF/KDR (R&D Systems, Abingdon, UK), 1 *µ*L APC-conjugated anti-CD133 (MACS Miltenyi Biotec, Bergisch Gladbach, Germany), and 0.5 *µ*L Pe-Cy7 conjugated mouse anti-human CD45 (BD Pharmingen). In order to minimize artifacts caused by dead cells, platelets, and erythrocyte fragments a “dump channel” was created as proposed by Mund et al. [29], which was used to exclude spurious events. Dead cells, platelets, and erythrocytes were stained by Sytox Blue dead cell stain (Invitrogen Molecular Probes, Bleiswijk, Netherlands), 3 *µ*L anti-human CD41a eFluor 450 (eBioscience, San Diego, CA, USA), and 3 *µ*L Pacific Blue anti-human CD235ab (HIR2 clone, 0.50 mg/mL) (BioLegend, San Diego, CA, USA), respectively. All stainings were performed in the presence of human FcR blocking reagent (MACS Miltenyi Biotec). CPCs were enumerated using a lyse-no-wash protocol, using counting beads as an internal reference to allow volumetric analysis. In order to minimize batch effects in antibody staining, ready-to-use tubes including all reagents were prepared at the onset of the study. Monoclonal antibodies and FcR blocking reagent were lyophilized in BD Trucount tubes (BD Biosciences, NJ, USA) and stored in the dark at 4°C before use.

On the day of blood withdrawal, 50 *µ*L of EDTA-anticoagulated whole blood was added to the tubes with antibodies and incubated in the dark at 4°C for 30 minutes. Following incubation, the blood samples were lysed with a nonfixing ammonium chloride lysis buffer for 10 minutes. 1 *µ*L 1 mM Sytox Blue dead cell stain (Invitrogen Molecular Probes, Bleiswijk, Netherlands) was added directly before analysis. CPCs were subsequently enumerated using a BD FACSCanto II flow cytometer (BD Biosciences). Acquisition was done until 30000 fluorescent beads were aspirated, which corresponds to a fixed volume of 30 *µ*L. Data analysis was performed by a blinded operator. Several types of CPCs were studied by flow cytometry: CD34+/CD45dim, CD34+/CD133+, CD133+, and CD34+/KDR+ subsets, where CD34+/CD45dim cells were considered to be the main CPCs and were identified by excluding events in the “dump channel,” gating for CD34+ cells and excluding CD45-bright cells (see Supplementary Figure 1 in the Supplementary Material available online at http://dx.doi.org/10.1155/2016/1487051).

### 2.5. Circulating Angiogenic Cells

The mononuclear cell fraction from blood was isolated by gradient density centrifugation. Whole blood was diluted in phosphate buffered saline containing 2 mM EDTA, carefully layered on top of Ficoll-Paque Plus (GE Healthcare), and centrifuged for 30 minutes at 400 ×g. Next, the number of cells was determined using Abbott Cell Dyn 1900 Hematology Analyzer, and cells were resuspended in endothelial growth medium (EGM-2, Lonza, Basel, Switzerland) containing SingleQuots® (Lonza) and supplemented with 10% fetal calf serum.

In order to allow accurate quantification, cells were placed in a cell-culture dish with a preetched counting grid (*µ*-Dish Squared 500x (Ibidi, Martinsried, Germany)) with a grid size of 0.25 mm^2^, coated with human fibronectin (1 *µ*g/mL) (Yo Proteins AB, Huddinge, Sweden). The cells were incubated in humidified incubator for 7 days at 37°C and 5% CO_2_ and received new medium on day 4. Uptake of acetylated low-density lipoprotein (AcLDL) was assessed by incubating cells with DiI conjugated AcLDL (2.5 *µ*g/mL, Invitrogen Molecular Probes) for 1 hour at 37°C. Cells were then fixed with 4% paraformaldehyde for 15 minutes at room temperature. Next, cells were incubated with FITC conjugated lectin from* Ulex europaeus *(2 *µ*g/mL) (Sigma, Saint Louis, MO, USA) for 1 hour at 4°C. Nuclei were visualized using Hoechst 33342 (10 *µ*g/mL) (Invitrogen). Cells that actively took up LDL and bound lectin to their surface receptors were considered to be circulating angiogenic cells (CACs). The number of CACs in three fields of 0.25 mm^2^ was counted using an internal grid (see Supplementary Figures 2A–C).

### 2.6. Anti-Müllerian Hormone

The AMH assay was performed with a sandwich ELISA (AMH Gen II ELISA, A79765, Beckman Coulter, Inc., USA). Assay buffer was added prior to analysis, in order to minimize complement interference. The lower limit of detection was 0.16 *µ*g/L. Interassay variation was 10% at 0.27 *µ*g/L and 4.7% at 3.9 *µ*g/L.

### 2.7. Additional Vascular Biomarkers

Multiplex immunoassays were carried out using the Bio-Plex System FLEXMAP 3D. The following proteins were measured: interleukin 6 (IL-6), IL-8, tumor necrosis factor *α* (TNF*α*), interferon gamma induced protein 10 (IP-10), stem cell factor (SCF), soluble SCF receptor (sSCF-R), vascular endothelial growth factor (VEGF), E-selectin, soluble intercellular adhesion molecule (sICAM), and soluble vascular cell adhesion molecule (sVCAM). Acquisition was performed with xPonent 4.2 and data analysis with Bio-PLex Manager Software version 6.1.1 (Bio-Rad Laboratories, Hercules, CA).

### 2.8. Statistical Analysis

The primary endpoint was the correlation between CD34+/CD45dim cells and AMH levels. Additional CPC subsets, CAC numbers, and vascular biomarkers (IL-6, IL-8, TNF*α*, IP-10, SCF, sSCF-R, VEGF, E-selectin, sICAM, and sVCAM) were determined and compared to AMH as a secondary outcome. All information was entered into SPSS (Statistical Package for Social Sciences) for Windows version 20 (SPSS Inc., Chicago, IL, USA) or R (R Foundation for Statistical Computing, Vienna, Austria). Baseline data were compared between patients and controls using a chi-square test for dichotomous data (i.e., current smoker, yes/no, current OC use, yes/no, and nulliparous, yes/no) and an independent samples two-tailed *t*-test for continuous data (i.e., age, BMI, duration of diabetes, and HbA1c value), with *α* of 0.05. For the analysis of the outcomes, a 2-way Analysis of Variance (ANOVA) was performed in order to correct for both diabetes status and OC use as binary variables and their interaction. The variables age, BMI, and current smoking status were considered to be potentially of influence on both endothelial function and AMH levels and were therefore additionally included in the multivariable analysis. Spearman rank correlations are given between continuous variables. Because the within-pair mean square error did not differ significantly from the residual mean square error in a one-way matched ANOVA analysis, a nonpaired 2-way analysis was performed, enabling the inclusion of all study participants for analysis. The current study was conducted as a pilot study for a correlation between CPCs and AMH, with insufficient available information for an* a priori* power calculation. As previous studies found a significant difference of CPC levels in groups of 15–20 participants [26–28], we therefore aimed to include at least 20 participants per study group, in order to potentially find statistically different CPC numbers.

## 3. Results

### 3.1. Study Population Characteristics

Twenty-two women with DM-1 and 20 healthy controls were included (see Table 1 for baseline characteristics). The mean age of all participants was 30.3 ± 8.2 years. All patients with DM-1 used short-acting insulin and four patients additionally used a long-acting insulin agent. The duration of diabetes varied between <1 and 38 years, with a median of 14.5 and mean duration of 14.8 ± 9.8 years. Glycemic control in the DM-1 group was suboptimal with a mean HbA1c value of 72.8 ± 16.0 mmol/mol (with a reference value of 53 mmol/mol for patients with diabetes). BMI, the proportion of nulliparous women, the number of pack years of smoking, and current smoking status did not significantly differ between the patient and reference group. Of all nulliparous participants, none had ever been or tried to become pregnant. For two DM-1 patients, it was not possible to find an age and OC matched healthy reference after inclusion. Two women could further only be matched according to age.

### 3.2. Correlations

**Figure 1:**
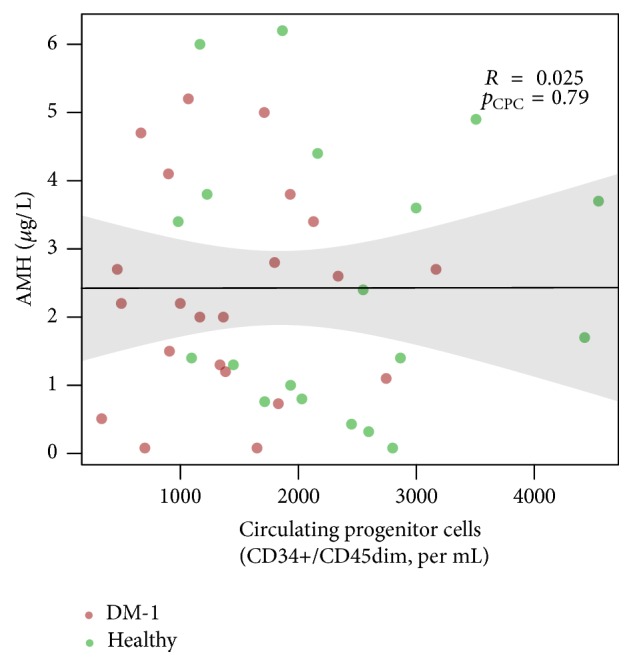
Correlation between AMH and number of CD34+/CD45dim cells. Number of CD34+/CD45dim cells was not correlated with AMH levels (crude *R* = 0.025, *p* = 0.79).

AMH levels were negatively correlated with subject age (*R* = −0.41, *p* = 0.007). Circulating CD34+/CD45dim cell levels were not associated with AMH levels in a multivariable analysis (adjusted beta = −0.0001, *p* = 0.78; unadjusted correlation coefficient *R* = 0.025, *p* = 0.79, Figure 1). There was not a significant interaction of diabetes status and OC use (*p* = 0.37). This lack of an association was not altered with the exclusion of OC users or smokers. None of the additional markers of vascular damage was furthermore related to AMH levels.

### 3.3. Circulating Progenitor Cells

**Figure 2:**
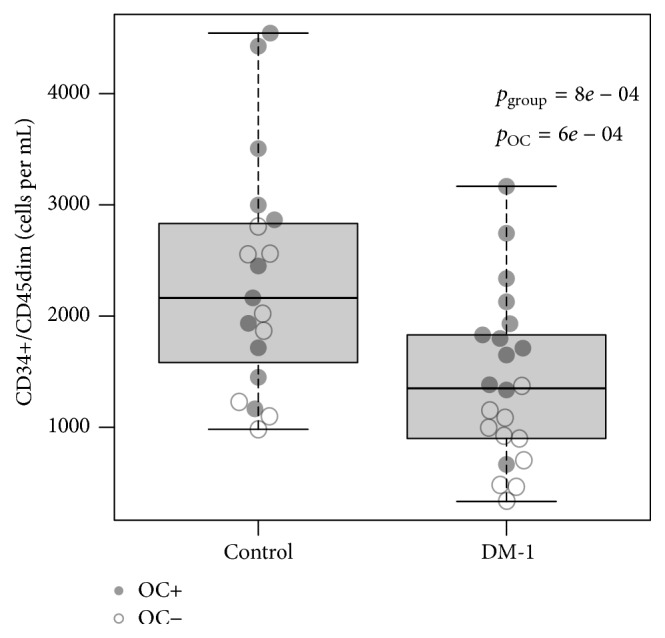
Boxplot of number of CD34+/CD45dim cells for both study groups. CD34+/CD45dim cells were significantly reduced in patients with DM-1 (*p* = 8 · 10^−4^). OC use was associated with increased numbers of CD34+/CD45dim cells, regardless of study group (*p* = 6 · 10^−4^).

CD34+/CD45dim cell counts were higher in women using OC in both study groups and did not differ significantly between smokers and nonsmokers. After adjustment for confounders, there were 1086 fewer CD34+/CD45dim cells/mL in the DM-1 compared to the reference group, which was statistically significant (Table 2, Figure 2). The relationship between the lower CD34+/CD45dim cell counts and DM-1 status was slightly more pronounced when the analysis was repeated in only the nonsmokers, with an adjusted difference of 1094 CD34+/CD45dim cells/mL, 95% confidence interval (CI) −1674 to −519, *p* = 0.01. When the analysis was repeated with only non-OC users this difference was 1233 CD34+/CD45dim cells/mL (95% CI −2293 to −174, *p* = 0.03). Similar results were observed for circulating CD133+ and CD34+/CD133+ cells, the numbers of which were significantly lower in patients with DM-1 and higher in patients using OC (1716 ± 958 versus 997 ± 555, *p* = 0.004, and 1138 ± 755 versus 598 ± 477, *p* = 0.006, resp.). Numbers of CD34+/KDR+ cells and CACs obtained after 7 days in culture were not statistically different between patients and controls.

### 3.4. Anti-Müllerian Hormone

**Figure 3:**
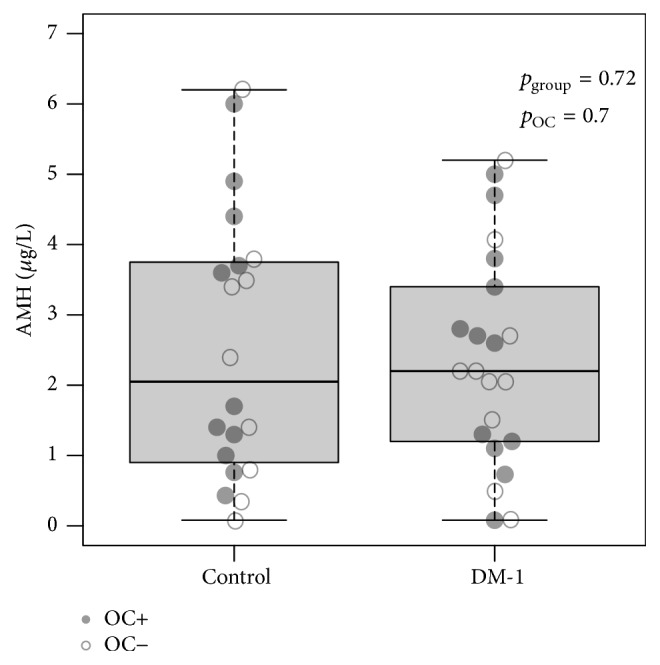
Boxplot of AMH levels for both study groups. AMH levels did not differ between patients and controls (*p* = 0.72) nor was there an effect of OC use (*p* = 0.70).

The median AMH levels [interquartile range] of the DM-1 and control group were 2.2 *µ*g/L [1.2–3.5] and 2.1 *µ*g/L [0.9–3.8], respectively (Table 2, Figure 3). AMH was nondetectable in two women with DM-1 and one control. One of the women with DM-1 had been using OC for 10 years; the other may have been approaching her perimenopausal stage based on her age of 46 years, despite regular menses. The participant in the control group with nondetectable AMH did not use OC but did have a BMI of >30 kg/m^2^. AMH was not related to HbA1c levels in the DM-1 group.

### 3.5. Additional Vascular Markers

Vascular damage markers sICAM and sVCAM were elevated in patients with DM-1 compared to healthy controls (see Table 2). Inflammatory markers IL-6, IL-8, and TNF*α* were detectable in only a few subjects. No differences were found between both groups for markers IP-10, SCF, SCF-R, and VEGF (see Supplementary Figure 3). Supplementary Figure 4 outlines the correlation between all studied variables.

## 4. Discussion

In this study, we did not find an association of early vascular damage markers with AMH in regularly cycling women. We demonstrated that several subsets of CPCs (CD34+/CD45dim, CD34+/CD133+, and CD133+) were reduced and sICAM and sVCAM were increased in patients with DM-1 compared to healthy controls, indicating the presence of early vascular impairment in this group of women with DM-1. AMH levels of patients with DM-1 were comparable to those in the reference group.

In conjunction with the absent relationship between macrovascular function parameters and AMH [20], we did not observe a relationship between microvascular function and AMH. This may suggest that the ovarian reserve of women with DM-1 is not affected by impaired vascular function. Interestingly, in a recent mice study, iatrogenic diabetes mellitus induction was shown to lead to a decrease in ovarian function [30]. It is possible that ovarian reserve could be influenced through other pathways of DM-1 pathophysiology, such as the formation of glycation end products as a result of hyperglycemia [31].

The similarity of AMH levels between women with DM-1 and controls is in line with the main finding of Soto et al. [15] and Yarde et al. [20]. This may in part be due to our inclusion of a relatively young study population, as Soto et al. did find a difference in AMH levels exclusively in the subgroup of women >33 years old (20 women with DM-1, 24 controls), regardless of cycle regularity [15]. Extrapolating from these results, it may be possible that any effect of DM-1 on ovarian aging, be it due to vascular factors or something else, may only occur at a later stage in life.

In our study population, more than 50% of the participants used OC. Several studies have suggested that AMH levels tend to be lower in OC users [32–34]. Furthermore, a novel and interesting finding of this study is the association between OC use and larger number of CPCs. This could be due to the role of estrogen, in accordance with previous findings that CPCs are increased in actively cycling women or women taking hormone replacement therapy compared to men or postmenopausal women [35, 36]. Moreover, changes in CPC numbers reflect fluctuations of estrogen during the menstrual cycle [37]. These observations may be explained by the induction of VEGF expression [38] and nitric oxide production by estrogen, leading to mobilization of CPCs from the bone marrow [39].

It stands to reason that in our study population vascular dysfunction was not present long enough or so severe as to cause a manifest reduction in ovarian reserve, considering the wide spread of duration of DM-1 and inclusion of a relatively young population with a mean age of 30 years. We did not assess manifestations of microvascular disease in the DM-1 group such as nephropathy or retinopathy and therefore unfortunately cannot relate ovarian reserve status or microvascular function to clinically present vascular complications. The finding of an average HbA1c value >53 mmol/mol in the DM-1 group cannot be readily clarified, as all patients reported insulin use and all attended secondary or tertiary specialist care. It is possible that this suboptimal glycemic control contributed to the signs of early vascular impairment observed in the DM-1 group.

To our knowledge, this is the first study investigating the relationship between AMH and markers of early vascular impairment in women with DM-1. Strengths in the design include the process of simultaneous DM-1 and control participant selection with respect to age and OC use, two important confounders. Performing blinded analyses of all outcome parameters and the use of standardized questionnaires eliminated the effects of observer bias. The small sample size of this pilot study poses a limiting factor for the interpretation and generalizability of our results. In addition, the cross-sectional design does not allow for causal interpretation, limiting its use to the observation of associations.

Despite the limitations, our study results are in line with earlier findings of a lack of an association between vascular function and ovarian reserve [40]. A post hoc power calculation furthermore illustrates the magnitude of sample size increase that would be necessary in order to potentially observe a correlation between early vascular damage markers and AMH, which would not be feasible to achieve. The results therefore suggest that there may not be an association between early vascular damage and ovarian reserve but should be interpreted with caution due to the limitations described above.

## 5. Conclusion

In this study we found that AMH, a marker of ovarian reserve, was not related to markers of early vascular dysfunction. We furthermore observed a reduced number of CPCs and increased levels of vascular damage markers in premenopausal women with DM-1 compared to healthy controls. A novel finding was the positive association of CPC number and OC use. From these results it can be concluded that adverse vascular endothelial conditions are present in young women with DM-1, and there are indications that this may not affect their ovarian reserve.

## Supplementary Material

The supplementary information contains the gating strategy utilized to identify circulating progenitor cells (CPCs).

## Figures and Tables

**Table 1:** Baseline characteristics.

	Diabetes mellitus	Healthy controls	*p* value
	*n* = 22	*n* = 20	
Age (years)	30.5 ± 8.2	30.2 ± 8.3	0.87
Oral contraceptive use	12 (54.5)	11 (55.0)	0.99
BMI (kg/m^2^)	24.0 ± 5.8	23.6 ± 4.0	0.76
Pack years of smoking	3.83 ± 6.2	0.95 ± 2.8	0.11
Current smoker	3 (13.6)	0 (0.0)	0.23
Nulliparous	14 (63.6)	12 (60.0)	0.81
Duration of diabetes (years)	15.1 ± 9.7		
HbA1c (mmol/mol)	72.8 ± 16.0		

Data presented as means ± SD or *n* (%).

*p* values represent the exact significance of a two-tailed *t*-test analysis for continuous data or a chi-square test for dichotomous variables.

**Table 2:** Outcomes for DM-1 versus healthy controls.

	Diabetes mellitus	Healthy controls	*p* value
	*n* = 22	*n* = 20	
AMH (*μ*g/L)	2.2 [1.2–3.5]	2.1 [0.9–3.8]	0.72
CD34+/CD45dim (/mL)	2007 ± 893	1350 ± 1069	<0.001
CD133+ (/mL)	1146 ± 912	1657 ± 745	0.04
CD34+/CD133+ (/mL)	649 ± 498	1164 ± 786	0.01
sICAM (ng/mL)	677 ± 144	560 ± 122	0.01
sVCAM (ng/mL)	2381 ± 613	1973 ± 559	0.02

Data presented as median [IQR] or mean ± SD.
