# Supplementary material for: Early Vascular Damage in Young Women with DM-1 and Its Relation to Anti-Müllerian Hormone: A Cross-Sectional Study
Source: Int J Endocrinol. 2016 Aug 29;2016:1487051. doi: 10.1155/2016/1487051 (PMC5019925; doi:10.1155/2016/1487051)
Supplement: Supplementary file 1 — The supplementary information contains the gating strategy utilized to identify circulating progenitor cells (CPCs). [file 1487051.f1.pdf]

Supplement

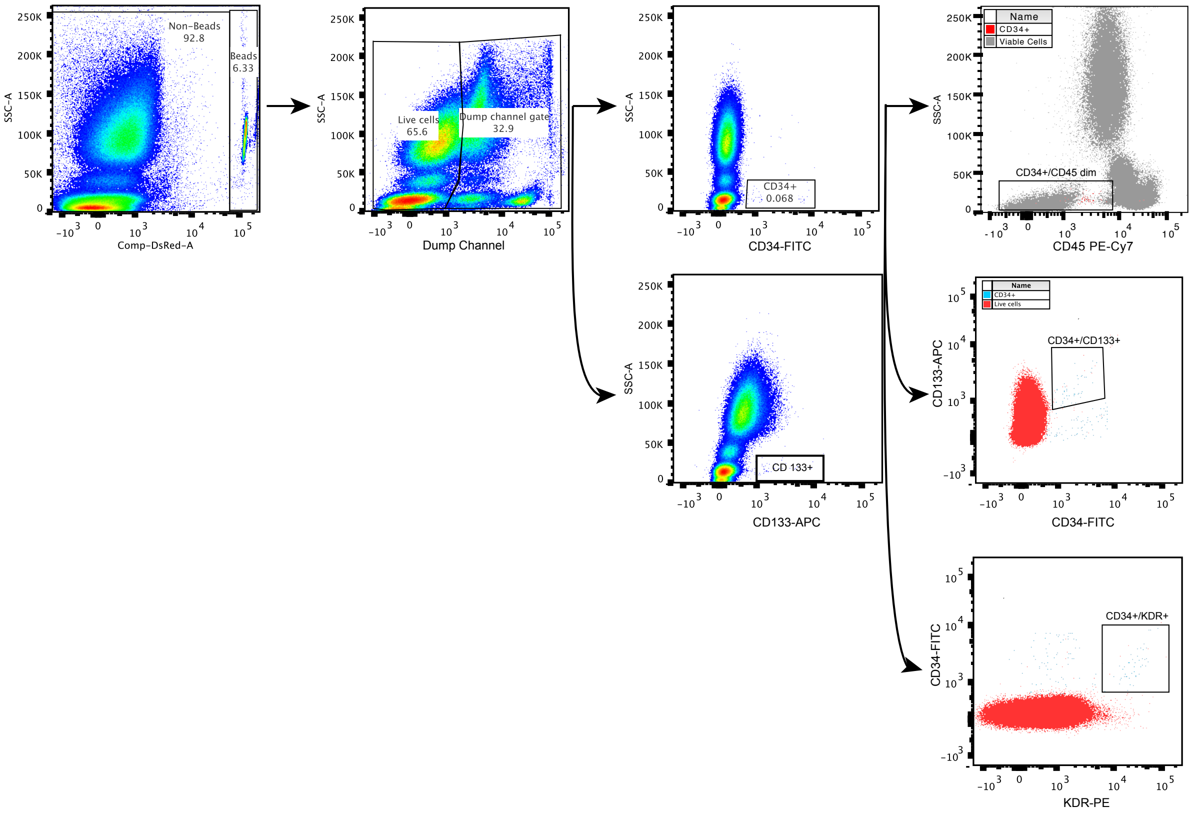

Figure 1: **Gating Strategy:** This figure shows the sequential hierarchical gating strategy employed in this paper. In the two leftmost panels first reference beads are counted and then unwanted events are excluded. Next CD34+ and CD133+ cells are identified within the live cell gate. From within the CD34+ gate, CD34+/CD45dim, CD34+/CD133+ and CD34+/KDR+ populations are identified. For clarity, populations are back-gated on top of the live cell gate.

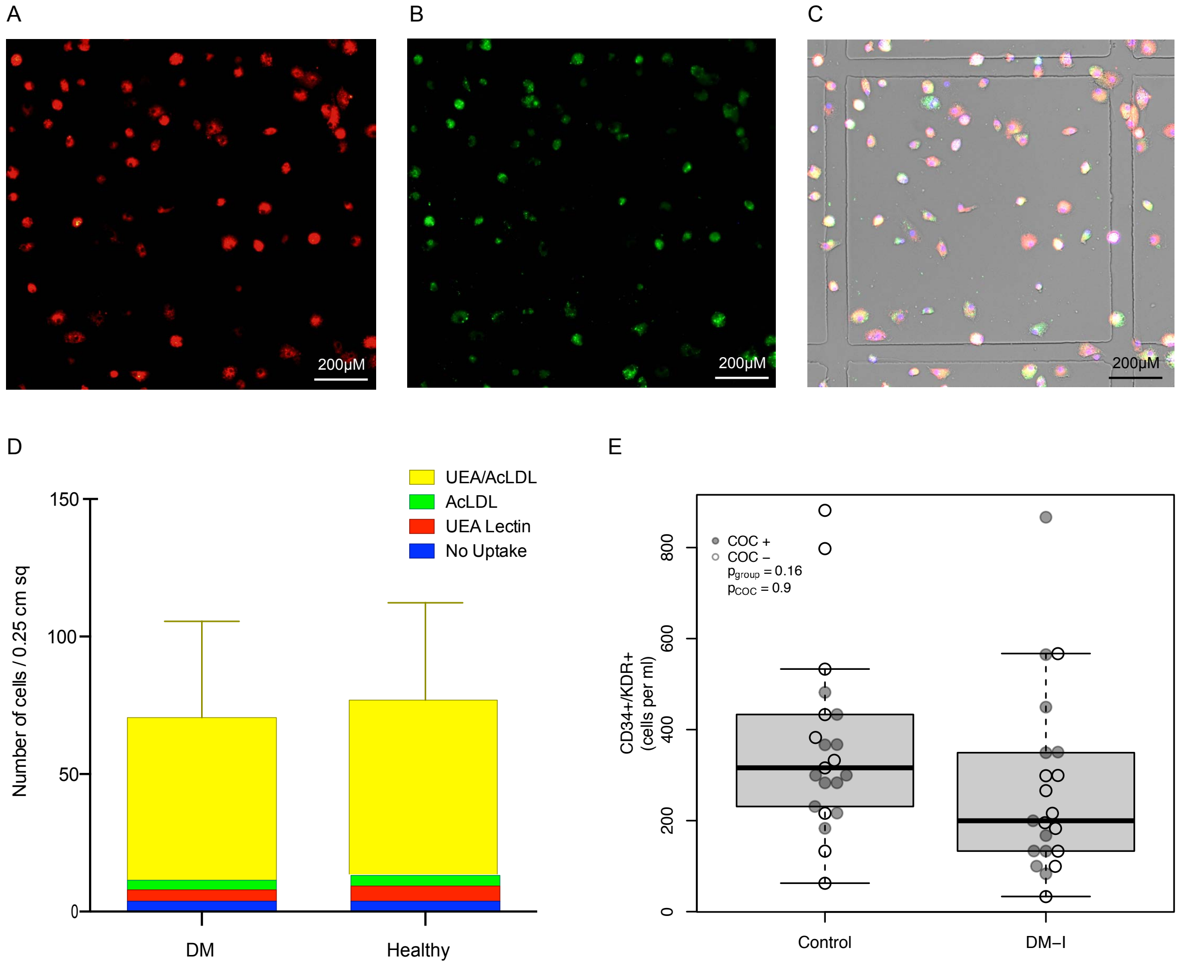

Figure 2: **Circulating Angiogenic Cells: A-C:** Representative example of AcLDL (**A**) and UEA staining (**B**), panel **C** shows the image overlay on the counting grid. **D:** Numbers of CACs or other attaching cells did not differ between patients with DM-I and controls. **E:** Numbers of Circulating CD34+/KDR+ cells as measured by flow cytometry in whole blood did not differ between patients and controls, nor was there an effect of OC use.

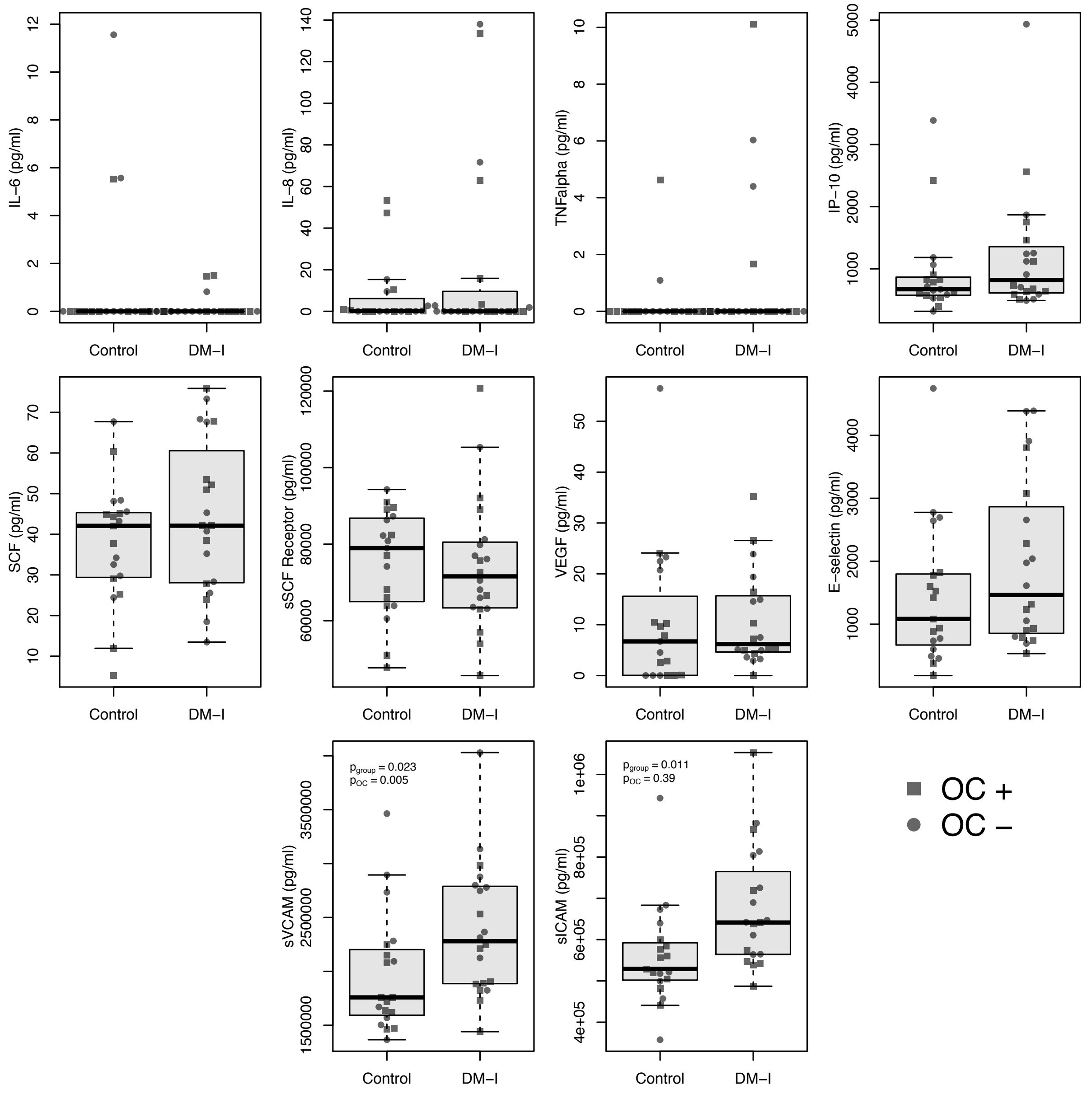

Figure 3: **Plasma markers:** Panels show levels of IL-6, IL-8, TNFα, IP-10, SCF, sSCF Receptor, VEGF, E-Selectin, sVCAM and sICAM in diabetics and controls. Squares indicate subjects using Oral Contraceptives (OC), circles indicate subjects not using OC. Significant differences between diabetics and controls were found for sVCAM and sICAM (p=0.023 and 0.011 respectively), a significant effect of OC was found in sVCAM levels (p=0.005).

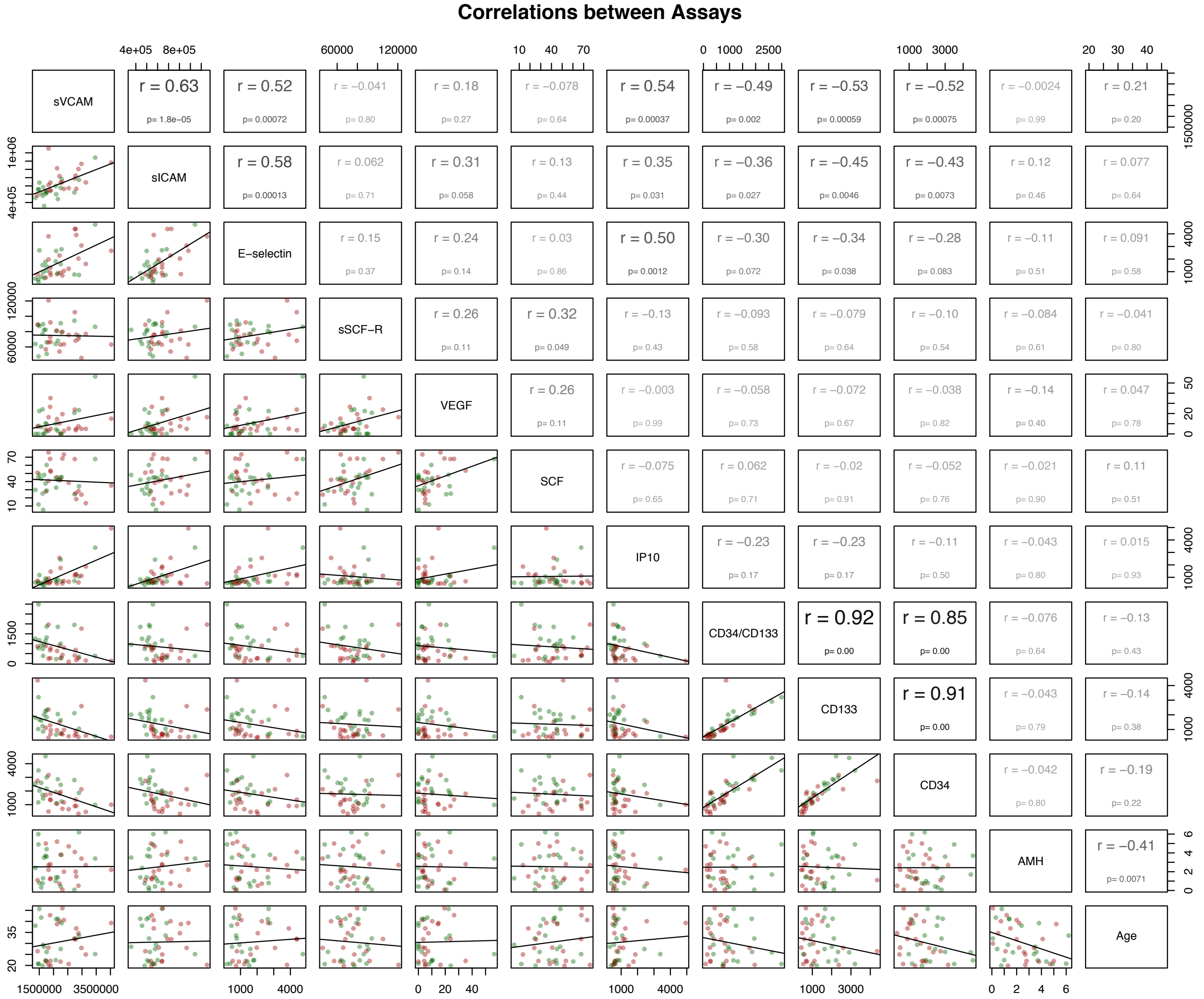

Figure 4: **Correlation Matrix:** Correlation matrix between age, protein markers and circulating cells as measured in this study. The upper right half shows spearmans ρ of a given pairwise comparison and the associated p-value (fontsize corresponds to effect size). The lower left half shows the bivariate scatterplot, DM-I patients are indicated in red, healthy controls in green. The data shows 3 prominent clusters: positive correlations between vascular damage markers (top left), strong positive correlations between the different progenitor cell subsets (right lower third) and an inverse relationship between vascular damage markers and circulating progenitor cells (top middle). Age is the only variable associated with AMH levels (bottom right).
